# Supplementary material for: Magnitude of risky sexual practice and associated factors among big construction site daily laborers in Bahir Dar city, Amhara Region, Ethiopia
Source: PLoS One. 2020 Oct 30;15(10):e0241220. doi: 10.1371/journal.pone.0241220 (PMC7598462; doi:10.1371/journal.pone.0241220)
Supplement: Attachment — Submitted filename: Response to Reviewers.docx [file pone.0241220.s003.docx]

**Response to Reviewer**

Dear Dr. Andrew R. Dalby (PhD)

Academic Editor, PLOS ONE

I and my colleagues are pleased to re-submit the revised version of our manuscript to you (Manuscript number: ***PONE-D-20-15399*** *and* Full title: ‘***Magnitude of Risky Sexual Practice and Associated Factors among Big Construction Site Daily Laborers in Bahir Dar City, Amhara Region, Ethiopia’***)

Thank you for giving us the opportunity to revise and re-submit. In keeping with your last message, I am re-submitting our revised manuscript within the stated deadline by rearranging the ethics statement the methods section.

I want to thank the reviewer for his/her detailed and educative comments and suggestions. We have incorporated the recommended changes in the manuscript to the best of our ability and we feel that our revised manuscript has been significantly improved as the comments of the reviewer were very excellent.

Our response is given specifically to each comment in the memo below and we have highlighted our changes in the main manuscript text. The reviewer’s comments are placed in the italic text, and our responses to the reviewer’s comments are listed just beneath each comment.

Thank you once again for spending your time and attention to our manuscript.

I look forward to work with you!

Best regards,

Amare Alamirew Aynie

Corresponding author

**Reviewer’s Comments and our Responses**

1. *The introduction section lacks coherence. For instance, the fourth paragraph should be before the third one.*

Thank you for your critical review!

We have re-arranged the paragraphs and made our introduction coherent (page 3 of our manuscript).

2. *You have mentioned that most studies in Ethiopia and abroad were mainly focusing on risky sexual behaviors; but the risky sexual practice was not well studied. Can you explain the difference between risky sexual behavior and risky sexual practice in your manuscript?*

Thank you!

Risky Sexual Behaviors are the behaviors of individuals’ that might lead to do Risky Sex. These include early sexual initiation, having multiple sexual partners, having sex partner outside marriage, watching pornographic movies, etc. While Risky Sexual Practices are practical acts that might put individuals’ health directly at risk; These may include unprotected sex with CSWs, unprotected sex outside marriage, unprotected transactional sex (unprotected sex for a wage), unprotected oral or/and anal sex, inconsistent condom use, etc.). (Please see page 4 of our manuscript).

3. *The justification for conducting this study is not clear. Please can you write something on what has been done so far by others in your country and beyond on similar or related topic. You only mentioned “The prevalence and its determinants of risky sexual behavior was documented before seven years by other investigators, but current risky sexual practice and its determining factors have not yet been explored.” To what extent are you confident to say current risky sexual practices have not been explored? Even if you are confident enough you must correct your statement by putting some uncertainty. Moreover, support what you wrote with references.*

Thanks a lot!

The major reasons for conducting this research are justified, references for the reviewed literatures are placed, and other important comments you raised have also been addressed. (Please see page 4 of our manuscript).

4. *Under methodology you have used proportion of risky sexual behavior as 44.9 %. Does it is proportion of risky sexual behavior among daily laborers? If not it should be changed to prevalence of RSB among daily laborers in your study area or other place. Otherwise you should take 50%.*

Thank you once again!

It is the prevalence of risky sexual behavior among day laborers; and it is taken from the previous study and cited by the reference number. (Page 5 of our manuscript).

5. *What was the rationale behind taking 20% of the big construction site? Please include under sampling technique and support with reference. In addition, you are expected to put the rationale for taking any variables with p-value of <0.2 on bivariate regression for multivariate analysis*.

Thank You!

- For the sampling purpose, we considered each of the 56 big construction sites as clusters. And as a simple rule of thumb, when using clusters for sampling it is advisable to include as many clusters as possible (up to 20%). This was our rational, and we put the supporting references. (Page 5 of our manuscript).
- The rational for taking *variables with p-value of <0.2 was to* control the possible effect of confounders. (This has been included on page 7 of our manuscript).

6. *Can you include inclusion and exclusion criteria for the study?*

Thank you!

We have done this as per the comment. (Page 6 of our manuscript).

7. *You have operationalized several terminologies (e.g. Big construction, risky sexual behavior, etc.). Have you defined these terminologies by yourself or you adopted definition given by someone or institution. If you adopted it, please write the references.*

Thanks a lot!

The definition of the terms were adopted from others, and cited in the reference. (Page 6 of our manuscript).

8. *Do you have other justification for disparity with previous studies in Ethiopia regarding prevalence of risky sexual practices other than the time of study and sample size? Please justify the disparities in scientific way. Hopefully there more reasons for the difference.*

Thank You for your in-depth sight!

As per the comments you provided, different possible justifications have been added to disparities of this study with the previous studies. Possible disparities included in the manuscript other than the time of study and sample size is:

- The difference in focused concept of study. The previous study was conducted on Risky Sexual Behaviors, while the present study has concerned only with Risky Sexual Practices.
- The difference in the focused population groups of the previous studies - most previous studies were conducted on high school, college and/or university students. Because of this, variables like the history of transactional sex were not included in the previous studies.
- The difference in the socioeconomic status of respondents and
- The difference in the living arrangement of respondents. (All these have been organized and written in a coherent way on Page 13 of our manuscript).

9. *With regard to factors associated with risky sexual practices instead of comparing your findings with previous similar studies you are expected to reason out why you got such findings.*

Thank You for your in-depth sight!

All factors that have a statistical association with risky sexual practice have been justified accordingly as per your constructive comments. (Please see Page 13-14 of our manuscript).

10. *Can you mention the new things your research brought to scientific community?*

Thank you in advance for your critical view!

In its overall content (particularly at its conclusion part), this study has shown two major new points to the scientific community and to the decision makers in Bahir Dar City.

- The first point is that, despite their high vulnerability to HIV infection, daily laborers are relatively forgotten (as to our review except few most investigators have given concerns for high school, preparatory and university students). Therefore, this research is powerful to click scholars to give due attention to these population groups, and it can also be voluble data for decision makers, so as to plan targeted prevention strategies in HIV/AIDS prevention.
- In the second place, though behaviors could potentially lead to practices, investigating Risky Sexual Practices could be more indicative to justify and estimate practical acts that might put individuals’ health directly at risk of HIV infection than investigating Risky Sexual Behaviors.

11. *Generally this manuscript need language improvement*

The language of the whole manuscript is edited using **Grammarly and Reverso Speller** online language editing software along with the language editing professional.
