# Supplementary material for: Magnitude of risky sexual practice and associated factors among big construction site daily laborers in Bahir Dar city, Amhara Region, Ethiopia
Source: PLoS One. 2020 Oct 30;15(10):e0241220. doi: 10.1371/journal.pone.0241220 (PMC7598462; doi:10.1371/journal.pone.0241220)
Supplement: Attachment — Submitted filename: Reviewer Comments.docx [file pone.0241220.s002.docx]

Reviewer Comments

1. The introduction section lacks coherence. For instance, the fourth paragraph should be before the third one.
2. You have mentioned that most studies in Ethiopia and abroad were mainly focusing on risky sexual behaviors; but risky sexual practice was not well studied. Can you explain the difference between risky sexual behavior and risky sexual practice in your manuscript?
3. The justification for conducting this study is not clear. Please can you write something on what has been done so far by others in your country and beyond on similar or related topic. You only mentioned “The prevalence and its determinants of risky sexual behavior was documented before seven years by other investigators, but current risky sexual practice and its determining factors have not yet been explored.” To what extent are you confident to say current risky sexual practices have not been explored? Even if you are confident enough you must correct your statement by putting some uncertainty. Moreover, support what you wrote with references.
4. Under methodology you have used proportion of risky sexual behavior as 44.9 %. Does it is proportion of risky sexual behavior among daily laborers? If not it should be changed to prevalence of RSB among daily laborers in your study area or other place. Otherwise you should take 50%.
5. What was the rationale behind taking 20% of the big construction site? Please include under sampling technique and support with reference. In addition, you are expected to put the rationale for taking any variables with p-value of <0.2 on bivariate regression for multivariate analysis.
6. Can you include inclusion and exclusion criteria for the study?
7. You have operationalized several terminologies (e.g. big construction, risky sexual behavior etc). Have you defined these terminologies by yourself or you adopted definition given by someone or institution. If you adopted it, please write the references.
8. Do you have other justification for disparities with previous studies in Ethiopia regarding prevalence of risky sexual practices other than the time of study and sample size? Please justify the disparities in scientific way. Hopefully there more reasons for the difference.
9. With regard to factors associated with risky sexual practices instead of comparing you findings with previous similar studies you are expected to reason out why you got such findings.
10. Can you mention the new things your research brought to scientific community?
11. Generally this manuscript need language improvement
